# Ultra-broadband Optical Gain Engineering in Solution-processed QD-SOA Based on Superimposed Quantum Structure

**DOI:** 10.1038/s41598-019-49369-6

**Published:** 2019-09-09

**Authors:** Hamed Goli Yousefabad, Samiye Matloub, Ali Rostami

**Affiliations:** 1 Quantum Photonics Research Lab (QPRL), University of Tabriz, Tabriz, 5166614761 Iran; 2 Photonics and Nanocrystals Research Lab (PNRL), University of Tabriz, Tabriz, 5166614761 Iran

**Keywords:** Optoelectronic devices and components, Quantum dots

## Abstract

In this work, the optical gain engineering of an ultra-broadband InGaAs/AlAs solution-processed quantum dot (QD) semiconductor optical amplifier using superimposed quantum structure is investigated. The basic unit in the proposed structure (QDs) is designed and fabricated using solution-processed methods with considerable cost-effectiveness, fabrication ease, and QDs size tunability up to various limits (0.1 nm up to the desired values), considering suitable synthesis methods. Increasing the number of QDs, the device can span more than 1.02 μm (O, C, S, and L bands) using only one type of material for all QDs, and is not restricted to this limit in case of using more QD groups. Also, it can manipulate the optical gain peak value, spectral coverage, and resonant energy for customized optical windows, among which 1.31 μm and 1.55 μm are simulated as widely-applicable cases for model validation. This makes the device a prominent candidate for ultra-wide-bandwidth and also customized-gain applications in general. Variation impact of homogeneous and inhomogeneous broadenings, injection current and number of QD groups on optical gain are explained in detail. Besides proposing a design procedure for implementation of an ultra-broadband optical gain using superimposed QDs in solution-processed technology, the proposed gain engineering idea using this technology provides practically infinite bandwidth and an easy way to realize. By introducing this idea, one more step is actually taken to approach the effectiveness of solution process technology.

## Introduction

Despite the ever-increasing development of optical communications, it is still suffering from the need for broader optical bandwidth^1^, especially in dense wavelength division multiplexing (DWDM) communications and high-speed computing systems. To resolve this issue, the operating bandwidth of optical components has to be improved. Among these, optical amplifiers have drawn great attention in recent years due to their numerous hybrid and integrated applications in optical networks^2^, silicon photonics^3^, all-optical switching^4^, and signal processing^5,6^, playing significant roles as amplifiers (preamplifiers, in-line amplifiers, and power boosters)^7–9^, wavelength converters^10–12^, modulators^13,14^, and all-optical logic gates^15^. Different types of optical amplifiers including solid-state^16^, doped fiber^17–19^, tapered^20^, Raman^21,22^, parametric^23–26^, and semiconductor optical amplifiers (SOAs)^27–31^ have been investigated for various output optical spectra.

In a study by Sun *et al*., optical amplifiers exploiting InN/GaN in their structure have reached optical gain bandwidths up to almost 1 μm for red to infrared, as a result of interband transitions between 50 minibands. However, their application has confronted problems due to the difficulty with fabrication techniques, integration with III-V compounds, and also high sensitivity to carrier densities at shorter wavelengths^32^. In another case, it has been reported that using SOA, an ultra-wideband microwave photonic filter (MPF) can span the free spectral range 15.44–19.44 GHz by managing the injection current in SOA; yet, there are still two major drawbacks associated with the proposed structure: firstly, fiber loop length applied in the structure is roughly 1.25 m which is considered very long in comparison with the cavity dimensions of multi-quantum well (MQW) SOA; second, the proposed structure demands extremely high injection currents (almost 200–500 mA) compared to the quantum dot SOAs (QD-SOAs)^33^. Although the structures containing power amplifiers based on transmission lines have high optical gain outputs (more than 20 dB), their optical gain bandwidth is limited over few GHz, and they are still incomparable with ultra-broadband QD-SOA in terms of optical gain bandwidth and device dimensions^34^. In another work, a power tandem single clad fiber amplifier was proposed to result in a broadband optical gain in 1900–2050 nm range. The major problems with this structure are its inapplicability for shorter wavelengths, low efficiency, and large dimensions compared to QD-SOAs^35^. Other proposed structures including fiber optical parametric amplifiers have reached broadband (over 1530–1600 nm) and high (more than 56 dB) optical gain values exploiting photonic crystal fibers. Nonetheless, these require the exploited fiber length to be more than 25 m and strictly depend on center pump wavelength in order to tune their optical gain bandwidth^36^.

Among different types of optical amplifiers, SOAs have been widely incorporated due to their ease of fabrication, cost-effectiveness, and compact size^37^. QD-SOAs, as one the promising types of SOAs, are of great interest because of their ultrafast gain recovery^38,39^, low threshold current, and temperature-insensitive operation^40^. In addition, their output gain characteristics are easily controllable by managing the QDs radii^41^.

Solution process nanotechnology is a simple and fast-growing technology to implement QDs and it is going to commercialize all related industrial products. In optoelectronics and photonics engineering, there are a lot of devices that can be implemented by this technology, but a broadband optical amplifier using this technology has not been reported. In these techniques, chemical synthesis methods are usually used to grow crystals with controlled sizes. Normally, the speed of growth can be controlled easily to obtain a precise size for synthesized QDs. Also, their shape and morphology can be tuned. On the other hand, there are some synthesis parameters such as concentration, temperature, pH, and the speed of rotation of solvent, by which the size of nanoparticles can be controlled. It should be mentioned that for low concentration cases if we consider that the most part of the colloid is solvent and a very small fraction is nanocrystals, very uniform and precise QDs will be available. If the high concentration is used, the size control will be very poor. It is shown that it is possible to synthesize nanoparticles with 0.1 nm size deviation. In poor synthesis conditions, the size resolution can be decreased even around the distribution mean value^42–48^.

InGaAs nanocrystalline QDs can be prepared from InCl_3_, Ga(NO_3_)_3_ and tris(trimethylsilyl)arsine (As[TMS]_3_)^49,50^. The reaction should be done in an inert gas like helium atmosphere, glove box or on a Schlenk vacuum line. For the synthesis of InGaAs, As[TMS]_3_ can be mixed with InCl_3_ in trioctylphosphine (TOP) and trioctylphosphine oxide (TOPO) at room temperature. The precursors should be injected into hot (300 °C) TOP in an inert atmosphere. The dot size can be controlled by growth time and a number of subsequent injections after the initial nucleation. Generally, longer growth times and increased precursor injections result in larger dots. This method normally gives a broad distribution of QD sizes, but in the case of low concentration of input material and slow reaction speed, it is possible to obtain a narrow distribution of nanoparticles. Meanwhile, Yu and coworkers have solved this problem with toluene/methanol to obtain narrower size distributions, too^50^. Additionally, it should be mentioned that with the electrophoresis method, one can separate the QDs of different sizes to make a narrow distribution. Thus, applying a normal synthesis method, it is possible to obtain a narrow distribution considering this method. Also, Hwang *et al*. claimed that particles of 5 nm size can be separated by this method^51^. Generally, the practical synthesis methods in this area are going to progress and obtain very high-resolution case.

**Figure 1:**
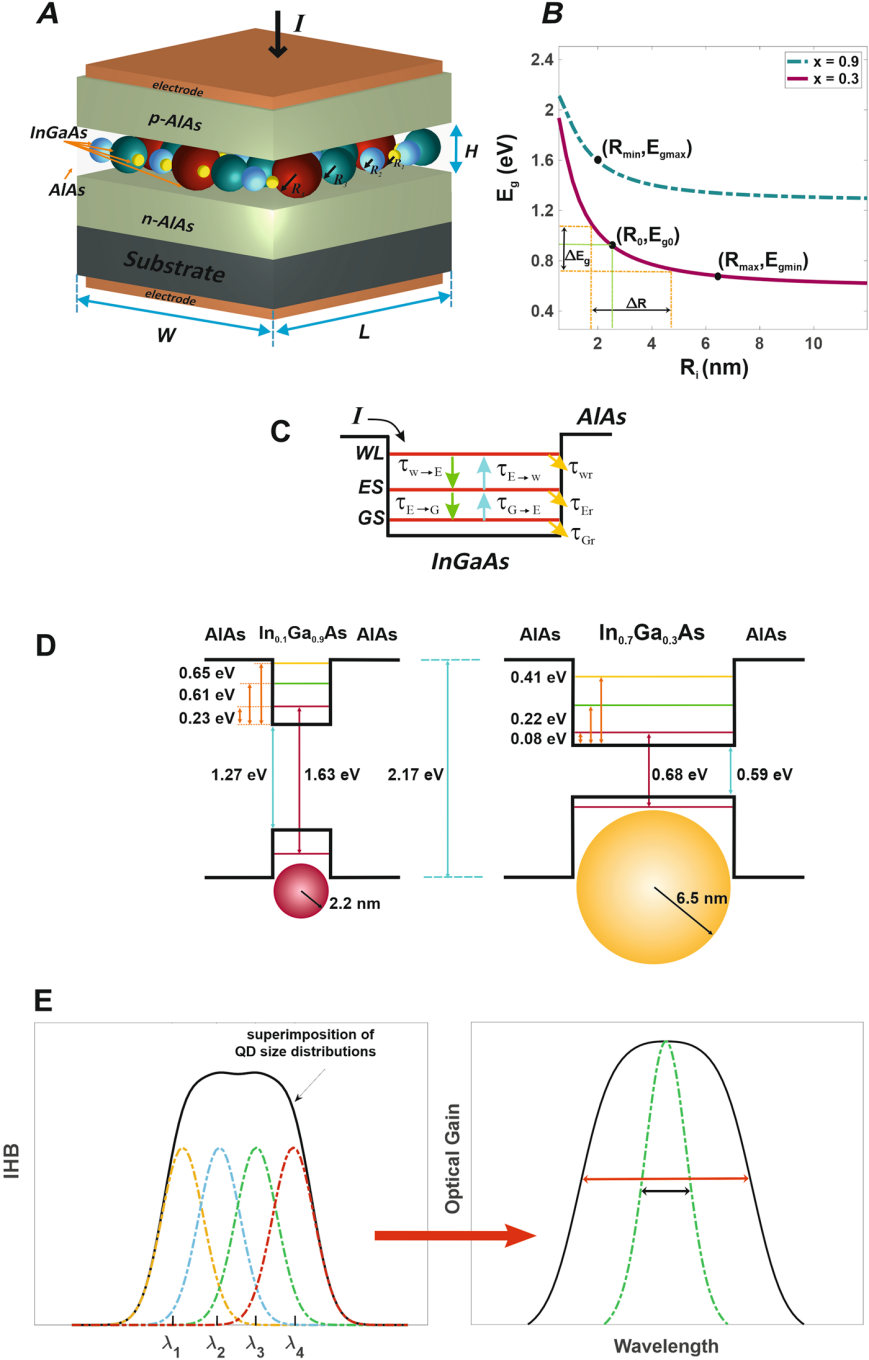
The proposed schematic structure, its band diagram, and optical gain spectrum broadening. (**A**) Schematic structure of the proposed QD-SOA. Different InGaAs QD groups are categorized based on their radii (*R*_1_ < *R*_2_ < *R*_3_ < *R*_4_), (**B**) the *E*_*g*_ − *R*_*i*_ diagram for the structure. This diagram illustrates bandgap energy versus QD radius distribution; two possible states are considered for In_1−x_Ga_x_As/AlAs structure with x = 0.3 (solid purple) and x = 0.9 (dash-dotted green). The minimum and maximum *E*_*g*_ values are 0.68 eV and 1.62 eV respectively for *R*_*i*_ = 6.5 nm and 2.2 nm, (**C**) the general band diagram for the proposed structure, (**D**) the band diagrams for the minimum and maximum radii of QDs in the structure, (**E**) schematic superimposition (solid black) of various QD size distributions (inhomogeneous broadenings (IHB)) over the radii *R*_1_ < *R*_2_ < *R*_3_ < *R*_4_ (respectively over corresponding *λ*_1_ < *λ*_2_ < *λ*_3_ < *λ*_4_, dash-dotted, left) results in broader optical gain profiles (right).

Last but not least, the proposed idea in this paper does not depend on the resolution of the synthesis method. Furthermore, the flat-band optical gain can be realized by high-resolution synthesis method, although there is a small oscillation in the passband of optical gain in low-resolution methods. To this end, a superimposed solution-processed QD-SOA modeling is proposed for InGaAs/AlAs structure in which several QD groups are categorized based on their size distribution. Controlling some factors such as the number, density and size of these QD groups besides working environment temperature, the structure demonstrates ultra-broadband and customized optical gain output which has strong potential to overcome limitations of previous works and could be utilized in numerous applications for wide spectral coverage. The proposed idea for wideband optical gain engineering is conceptually illustrated in Fig. 1. It is shown that using the superimposition of QDs, the optical gain with specific bandwidth and gain level is implemented. Also, as it is mentioned in the abstract, the QDs are synthesized by solution-processed methods and it is so easy to implement the idea. Finally, in this work, a new way is introduced to implement broadband optical amplifiers (practically infinite) and customized gain profile for different optical engineering applications.

## Concept and Modeling

Our model is proposed for InGaAs QDs confined in AlAs quantum well and synthesized by solution process methods. Incorporating these methods not only guarantee cost-effectiveness and simplicity for QD synthesis, but their radii could be easily controlled so that they can have variable distributions (from 0.1 nm to the desired values) over the mean radius of each QD group, depending on the desired accuracy and application. Figure 1(A) depicts the overall schematic structure for our proposed QD-SOA, including various QDs in its active region. One can realize from Fig. 1(B) that *E*_*g*_ − *R*_*i*_ diagram relates to bandgap energy versus QD size distribution based on which the resonant energies and size distributions for all QD groups are determined; this diagram is plotted as a result of numerically solving the Schrödinger equation for InGaAs/AlAs structure. The corresponding band diagram is shown as well in Fig. 1(C) indicating three sections including wetting layer (WL), excited (ES) and ground (GS) states considered and the lifetime constants necessary for carrier dynamics investigation. Figure 1(D) illustrates the band diagram for minimum and maximum QD radii (respectively as 2.2 nm and 6.5 nm), and superimposition (left, solid black) of various four QD size distributions (left, dash-dotted) is shown in Fig. 1(E). Also, the broadened optical gain spectrum for four QD groups (right, solid black) compared to that of one group (right, dash-dotted green) is illustrated in this figure.

**Figure 2:**
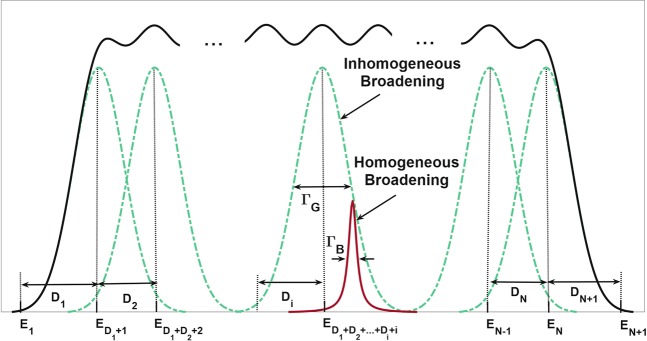
Our superimposed model for multiple QD groups. The proposed superimposition model in which *Γ*_*B*_ and *Γ*_*G*_ respectively denote the FHWM for homogeneous (solid purple) and inhomogeneous (dash-dotted green) broadenings. The widest profile (solid black) indicate superimposition of multiple Gaussian profiles for various QD groups. Resonant energies (*E*_*i*_) and the separations of IHB peak values (*D*_*i*_) are also included in the diagram.

Although QDs are synthesized with highly precise techniques, all will vary in size compared to each other due to fabrication and practical constraints. In our model, they are categorized into *N* groups containing a specific number of QDs with total size distribution over a specific *R*_0_. This size distribution imposes an inhomogeneous broadening (IHB) for every QD group and is assigned a Gaussian profile, as in Fig. 1(E). Therefore, QDs in the same color (in Fig. 1(A)) belonging to one specific group will result in their corresponding Gaussian profile (Fig. 1(E)); i.e., the profiles will be over *λ*_1_ < *λ*_2_ < *λ*_3_ < *λ*_4_ respectively resultant from QDs with *R*_1_ < *R*_2_ < *R*_3_ < *R*_4_ radii. The FWHM for each IHB is denoted as *Γ*_*G*_ (likewise with Fig. 2). As with Fig. 1(E), it is expected that superimposition of different IHBs (dash-dotted Gaussian profiles) for multiple QD groups causes optical gain spectrum (solid black) to experience some broadening over their specific energies.

Furthermore, due to inherent effects (e.g., temperature or pressure) on all QDs, they experience another type of broadening, known as homogeneous broadening (HB), as in Fig. 2; this broadening homogeneously impacts all groups and is assigned a Lorentzian profile with Γ_*B*_ as its FWHM (solid purple).

## General Formalization

As mentioned above, the radius of QDs can be managed in order to produce optical gain output at specific frequencies; i.e., a QD with a specific radius *R*_0_ will produce gain output at its corresponding energy *E*_0_. Thus, one can obtain broadband optical gain spectrum by simultaneously using several QDs with various values of *R*_0_. This broadening behavior underlies our superimposed model in which QDs are engineered so that their broadening profiles will lead to ultra-broadband optical gain spectrum. As one can see in Fig. 2, the resonant energy for each group at which gain output reaches its peak value is:

1 $${E}_{{M}_{i}}={E}_{{D}_{1}+{D}_{2}+\ldots +{D}_{i}+i}$$

where *D*_*i*_ refers to the energy separation between two subsequent resonant energies. Due to possible distinct overlaps between various IHBs (different separations between gain peak values of numerous QD groups), it has to be considered not the same for all groups; consequently, the model contains unique values of *D*_*i*_ for different subsequent ${E}_{{M}_{i}}\,\,$ values. The energy separation between any two subgroups, denoted by Δ*Ε*, is calculated as:

2 $$\Delta E=\frac{{E}_{{M}_{N}}-{E}_{{M}_{1}}}{{\sum }_{i=2}^{N}\,{D}_{i}}$$

The QD groups are all divided into *N*_*sub*_ subgroups:

3 $${N}_{sub}=(N-1)+\mathop{\sum }\limits_{i=1}^{N}\,{D}_{i}$$

The energy of each subgroup is denoted as *E*_*n*_ (*n* = 1, 2, …, *N*_*sub*_), and it will have its gain output at the corresponding mode (which will be assigned the subscript *m* later). The contribution of each QD subgroup to the IHB of its corresponding group can be generally written as follows:

4 $${G}_{i}=\frac{1}{\sqrt{2\pi \xi }}\exp [\frac{-{({E}_{n}-{E}_{{M}_{i}})}^{2}}{2{\xi }^{2}}]$$

where $\xi =\frac{{\Gamma }_{G}}{2.35}$ is QD coverage. Then, the total IHB for the superimposed model will be:

5 $${G}_{tot}=\mathop{\sum }\limits_{i=1}^{N}\,{G}_{i}$$

Moreover, the HB for each QD subgroup can be obtained as:

6 $${B}_{n}^{m}=\frac{\hslash {\Gamma }_{B}/\pi }{{({E}_{m}-{E}_{n})}^{2}+\,{(\hslash {\Gamma }_{B})}^{2}}$$

where ${B}_{n}^{m}$ is the HB for nth subgroup (and mth mode) and *E*_*m*_ refers to the output energy for mth mode.

## Rate Equations

In order to investigate carrier dynamics for the proposed structure along a specific time interval (*t*) and length (*z*) in the cavity, one has to describe the rate equations which are written for our model as the following:

7 $$\frac{\partial {N}_{w}(t,z)}{\partial t}=\frac{I}{qV}+\sum _{n}\frac{{N}_{Q}{h}_{n}(t,z)}{{\tau }_{E\to w}}-\frac{{N}_{w}(t,z)}{{\tau }_{wr}}-\sum _{n}\frac{{N}_{w}(t,z)[1-{h}_{n}(t,z)]}{{\tau }_{w\to E}}{G}_{tot}$$

8 $$\begin{array}{c}\frac{\partial {h}_{n}(t,z)}{\partial t}=\frac{{N}_{w}(t,z)[1-{h}_{n}(t,z)]}{{N}_{Q}{\tau }_{w\to E}}{G}_{tot}+\frac{{f}_{n}(t,z)[1-{h}_{n}(t,z)]}{{\tau }_{G\to E}}-\frac{{h}_{n}(t,z)}{{\tau }_{E\to w}}\\ \,\,\,\,\,\,-\frac{{h}_{n}(t,z)[1-{f}_{n}(t,z)]}{{\tau }_{E\to G}}-\frac{{h}_{n}(t,z)\,}{{\tau }_{Er}}\,\end{array}$$

9 $$\begin{array}{c}\frac{\partial {f}_{n}(t,z)}{\partial t}=\frac{{h}_{n}(t,z)[1-{f}_{n}(t,z)]}{{\tau }_{E\to G}}-\frac{{f}_{n}(t,z)[1-{h}_{n}(t,z)]}{{\tau }_{G\to E}}-\frac{{f}_{n}^{2}(t,z)}{{\tau }_{Gr}}\\ \,\,\,\,\,-\frac{\Gamma }{{N}_{Q}A}\,\sum _{m}\frac{{g}_{n}^{m}{P}_{m}(t,z)}{{E}_{m}}\end{array}$$

Here, *I* is the injected current which acts as the continuous electrical pumping source (i.e., it is applied constantly in the whole simulation time interval) and is responsible for the population inversion of carries in the structure, *q* is electron charge, and *V* denotes the active region volume (*V* = *H* × *W* × *L* as in Fig. 1(A)). Also, *N*_*Q*_ and *N*_*w*_ respectively indicate QD volume density and carrier density in the wetting layer (WL). If the former increases, the number of QDs in WL will reach higher values leading to more energy states for carrier occupation and higher optical gain values. With an increase in the latter, the effect would be the same because more carriers can relax from WL to QD energy states, which again causes higher optical gain outputs. Lifetime constants *τ*_*w*→*E*_, *τ*_*E*→*G*_, *τ*_*wr*_, *τ*_*Gr*_,*τ*_*E*→*w*_, and *τ*_*G*→*E*_, relate to relaxation from WL to ES, relaxation from ES to GS, recombination in WL, recombination in GS, reexcitation from ES to WL, and reexcitation from GS to ES. The values exploited for these constants are all listed in Table 1. The last term of Eq. (8) is negligible due to its large lifetime constant (*τ*_*Er*_).The confinement factor is described as *Γ*, and *A* is the cross-section for the active region (*A* = *H* × *W*). In the equations, *h* and *f* respectively indicate occupation probabilities for ES and GS. The modal gain for mth mode (resultant from nth subgroup) is calculated as:

10 $${g}_{n}^{m}={g}_{max}[2{f}_{n}(t,z)-1]{G}_{tot}{B}_{n}^{m}$$

where *g*_*max*_ is the maximum gain coefficient (gain saturation) and *f*_*n*_ is the occupation probability for the GS of nth QD subgroup. *P*_*m*_ and *E*_*m*_ respectively refer to the output power and energy of mth mode.

**Table 1:** Simulation parameters for the proposed model.

Symbol	Value	Description
*n*_*g*_	3.5^53^	refractive index for active region
*L*	1.5 [mm]	SOA length
*H*	0.25 [μm]^39^	height of active region
*W*	4 [μm]^39^	width of active region
*Γ*	1	confinement factor
*N*_*Q*_	2 × 10^21^ [1 /m^3^]^12,39^	volume density of QDs
*τ*_*wr*_	0.2 [ns]^1,39^	recombination lifetime for WL
*τ*_*w*→ *E*_	3 [ps]^12,39^	relaxation lifetime from WL to ES
*τ*_*E*→ *w*_	1 [ns]^12,39^	reexcitation lifetime from ES to WL
*τ*_*E*→ *G*_	0.16 [ps]^12,39^	relaxation lifetime from ES to GS
*τ*_*G*→ *E*_	1.2 [ps]^12,39^	reexcitation lifetime from GS to ES
*τ*_*Gr*_	0.4 [ns]^12,39^	recombination lifetime for GS
*α*_*int*_	0 [1 /m]^31,39^	material absorption coefficient
*g*_*max*_	14 [1/m]^1,39^	maximum gain coefficient
*I*	50 [mA]	injection current
*P*_*in*_	100 [µW]	amplitude of input power
${N}_{{w}_{0}}$	1 × 10^15^ [1/m^3^]	initial condition for WL
*h*_0_	0.8	initial condition for ES
*f*_0_	0.9	initial condition for GS

The propagation equation for the input signal of mth mode along the cavity (in *z* orientation) is:

11 $$\frac{\partial {P}_{m}(t,z)}{\partial z}=[\Gamma \sum _{n}\,{g}_{n}^{m}-{\alpha }_{int}]\,{P}_{m}(t,z)$$

where *α*_*int*_ is the material absorption coefficient.

Finally, total gain along the cavity can be described as:

12 $$G=10log[exp({\int }_{0}^{L}\,\sum _{m}\,\sum _{n}\,{g}_{n}^{m}dz)]$$

where *L* is the cavity length as in Fig. 1(A).

## Gain Engineering

**Figure 3:**
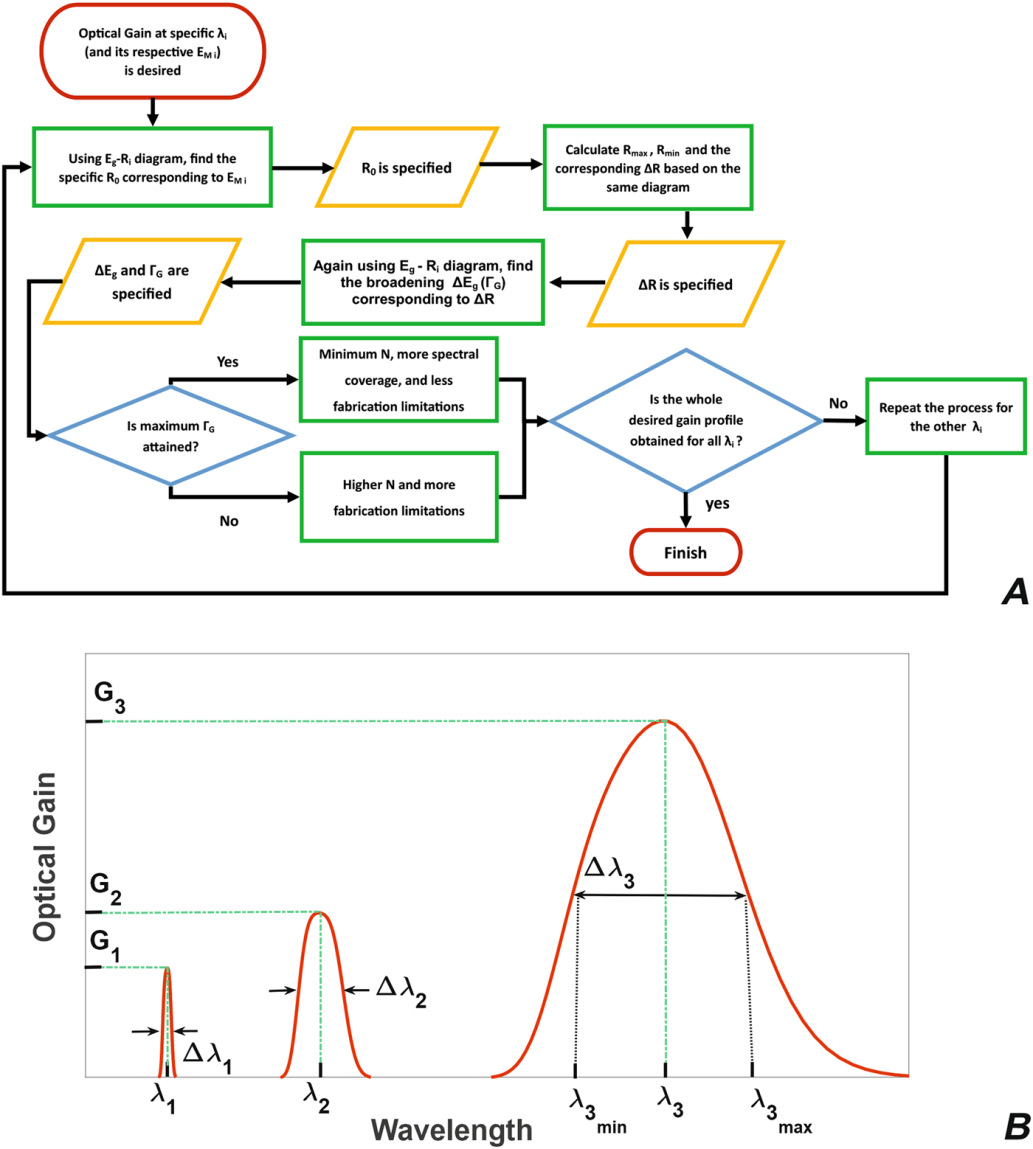
Flowchart of the proposed model and a schematic optical gain spectrum. (**A**) The main procedure of our model required for *ΔR*, *Γ*_*G*_, and *N*  calculation, (**B**) customized optical gain spectrum containing three peak values *G*_1_, *G*_2_, and *G*_3_ respectively over *λ*_1_, *λ*_2_, and *λ*_3_ wavelengths in Δ*λ*_1_, Δ*λ*_2_, and Δ*λ*_3_ regions (the respective FWHM values for these regions are ${\Gamma }_{{G}_{1}}$, ${\Gamma }_{{G}_{2}}$, and ${\Gamma }_{{G}_{3}}$).

Previously stated, although size distribution of QDs can be controlled for specific applications exploiting precise fabrication techniques, they still impose some limitations on the gain characteristics of SOA structure. With the size of QDs restricted, IHB can take specific values as *Γ*_*G*_ so that the maximum size distribution over a specific radius *R*_0_, denoted as Δ*R*_*max*_, gives the maximum *Γ*_*G*_. Following this result, one can figure out the minimum *N* necessary to produce the desired ultra-broadband gain output. As Fig. 3(A) depicts, over a specific ${E}_{{g}_{0}}$ (equal to ${E}_{{M}_{i}}\,\,$of that specific group), its corresponding *R*_0_ can be found using the *E*_*g*_ − *R*_*i*_ diagram in Fig. 1(B). The next step is the calculation of Δ*R* based on the maximum and minimum radii, *R*_*min*_ and *R*_*max*_ for each group (Δ*R* = *R*_*max*_ − *R*_*min*_) and its resultant Δ*E*_*g*_ (or *Γ*_*G*_) in accordance with the same diagram. Then having *Γ*_*G*_, the required *N* will be easily obtained.

Our flexible model can be managed in order to produce customized gain outputs; i.e., the structure will have optical gain output in some wavelength regions, leaving the others with no optical gain. Among these cases, a schematic instance is evidently seen in Fig. 3(B). This gain output encompasses three gain peak values, *G*_1_, *G*_2_, and *G*_3_ over *λ*_1_, *λ*_2_, and *λ*_3_ wavelengths for which the gain spectrum spans three various regions, Δ*λ*_1_, Δ*λ*_2_, and Δ*λ*_3_. The maximum and minimum desired wavelengths for Δ*λ*_1_ are denoted as ${\lambda }_{{1}_{max}}$ and ${\lambda }_{{1}_{min}}$ (${\lambda }_{{2}_{max}}$, ${\lambda }_{{2}_{min}},\,{\lambda }_{{3}_{max}}\,{\rm{and}}\,{\lambda }_{{3}_{min}}$likewise with the other two regions). In order to produce the desired gain spectrum for *λ*_1_, one has to follow the procedure in Fig. 3(A) firstly by finding the minimum, mean, and maximum radii in Δ*λ*_1_ region corresponding to ${\lambda }_{{1}_{min}}$, *λ*_1_, and ${\lambda }_{{1}_{max}}\,$using the *E*_*g*_ − *R*_*i*_ diagram. Then, Δ*R*_1_ can be simply calculated for this region which determines ${\Gamma }_{{G}_{1}}$ and subsequently the required *N*_1_ for the first region. The same procedure has to be followed for the other regions so as to produce the whole desired optical gain output, as the figure indicates. In addition, the proposed model is capable of tuning three major factors related to the customized optical gain spectrum: *λ*_*i*_ is manageable by changing QD size, IHB FHWM can be controlled by Δ*R*_*i*_, and finally the gain peak value (*G*_*i*_) varies by *N*_*i*_ and QDs volume density. Therefore, one could change different features of the structure to obtain the desired optical gain output with predetermined characteristics.

## Results and Discussion

The parameters applied in our simulations including structural parameters, lifetime constants, and initial conditions are mentioned in Table 1. Also, *Γ*_*G*_ is often set to 30 meV because 10% size variation over *R*_0_ = 2.2 nm results to almost 30 meV broadening over its corresponding resonant energy in accordance with the *E*_*g*_ − *R*_*i*_ diagram.

### Broadening effect considering multiple groups

**Figure 4:**
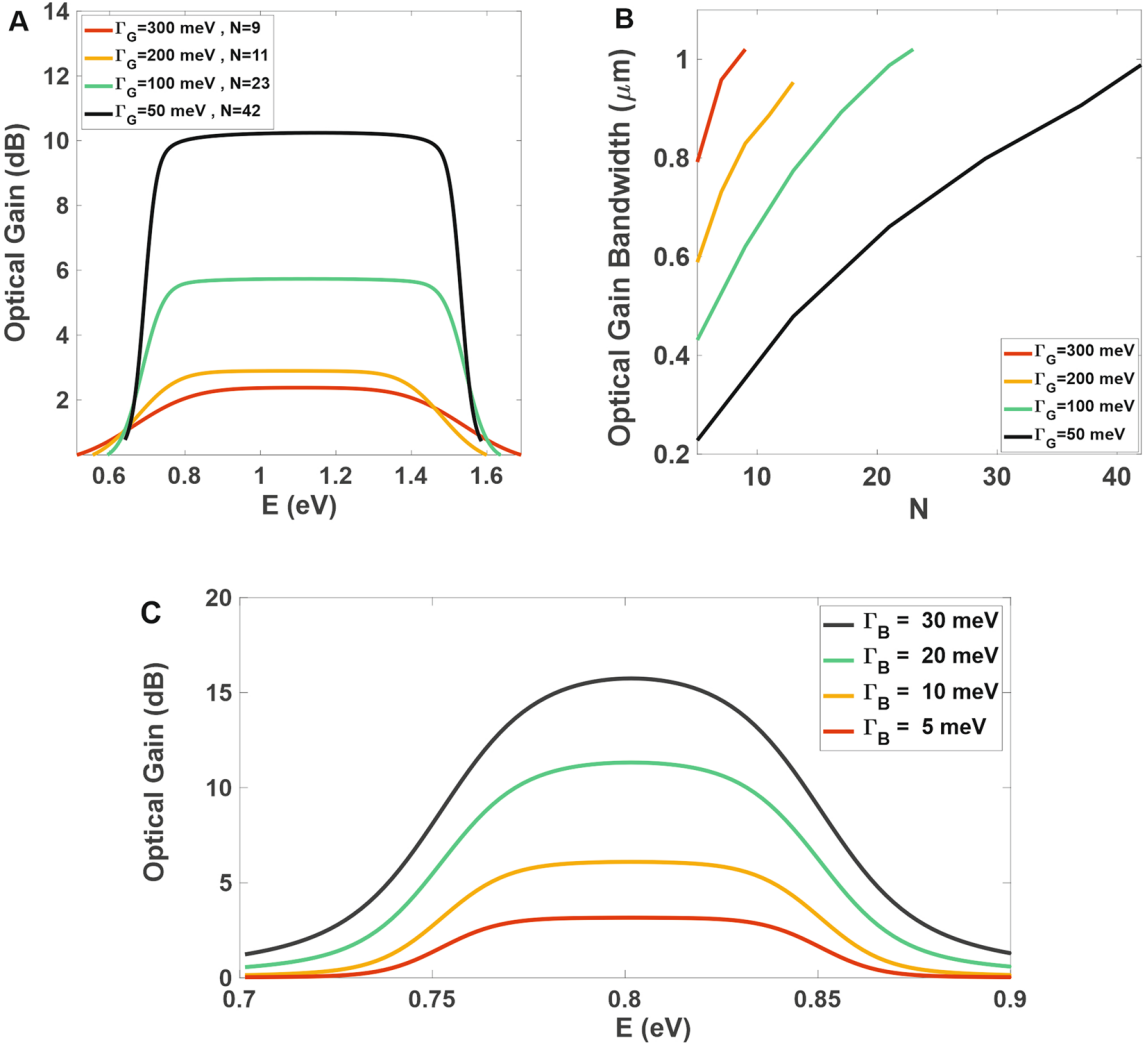
Effects of FWHM for inhomogeneous and homogeneous broadenings and number of QD groups on the optical gain. (**A**) Gain spectrum for *Γ*_*G*_ = 50, 100, 200, and 300 meV respectively with *N* = 42, 23, 11, and 9. The optical gain bandwidths obtained are respectively 0.81 μm–1.79 μm (black), 0.8 μm–1.82 μm (green), 0.86 μm–1.74 μm (orange), and 0.8 μm–1.82 μm (red)), (**B**) increase in optical gain bandwidth by changing the number of QD groups (N from 3 to 42) for those *Γ*_*G*_ values, (**C**) optical gain spectrum for *Γ*_*B*_ = 5, 10, 20, and 30 meV.

Broadening values are of great importance for our simulations as their FWHM can manipulate various QD characteristics. As mentioned above, *N* and consequently the bandwidth and peak value of optical gain strongly depend on IHB FWHM. Figure 4(A) depicts the change in optical gain considering *Γ*_*G*_ = 50, 100, 200, and 300 meV. Also indicated in the figure are various *N* (respectively as 42, 23, 11, and 9) corresponding to these *Γ*_*G*_ values which are found by the *E*_*g*_ − *R*_*i*_ diagram. As *Γ*_*G*_ accumulates, Δ*R* experiences some increase for each group, which requires more groups to be superimposed in our model. This accumulation of *N* yields an increase in the contribution of QD groups to the total IHB so that it needs to be shared between these groups, which is the main reason for its peak value decline. Conversely, with *Γ*_*G*_ restricted to small values, IHB gets narrower for each QD group and is just restricted to some of them; this guarantees more precision for optical gain over a specific wavelength (the resonant mode) and less superimposition effect of other groups.

Furthermore in Fig. 4(A), it can be seen that the maximum optical gain drops as a result of *Γ*_*G*_ increase. Here, *Γ*_*B*_ = 20 meV at room temperature (*T* = 300 K)^52^ which is in a good agreement with experimental results. Typically, we have set *Γ*_*G*_ = 30 meV which is considerable to *Γ*_*B*_ = 20 meV, but as it accumulates, its gain-dropping effect dominates *Γ*_*G*_ which in turn causes the mentioned decline. As in this figure, we have reached almost 1.02 μm optical gain bandwidth for only one type of material and *Γ*_*G*_ = 50 meV (and broader bandwidth even for higher *Γ*_*G*_), but it compromises the maximum optical gain. Figure 4(B) illustrates the optical gain bandwidth for multiple groups (different N) indicating that by increasing *N* or *Γ*_*G*_ the bandwidth meets higher values, the fact that conforms to Fig. 4(A).

On the other hand, Fig. 4(C) indicates one can change the HB FHWM and observe that it affects the optical gain output by homogeneously concerning all QDs and assisting their involvement in resonant modes. It is shown for *Γ*_*B*_ = 5, 10, 20, and 30 meV that by increasing *Γ*_*B*_, more QDs contribute to the resonant mode, which causes higher optical gain peak values.

### Maximum gain change with injection current for multiple groups

**Figure 5:**
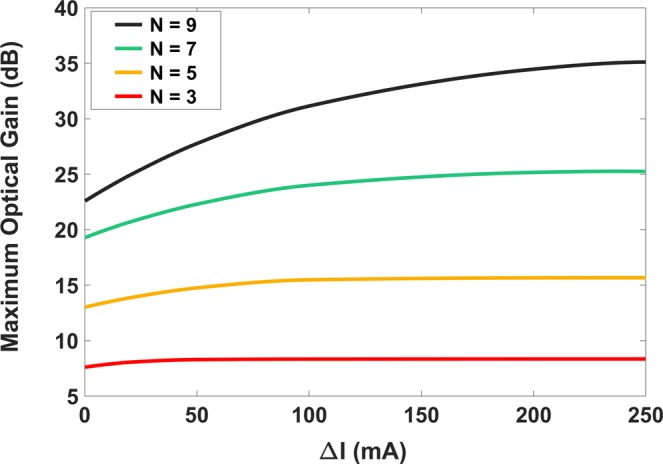
Impact of injection current on the optical gain. Maximum optical gain variation versus different injection current values for various numbers of QD groups (*N* = 3, 5, 7, 9). Note that horizontal axis is in terms of *ΔI* = *I* − *I*_0_.

As another impact, the optical gain variation with injection current has been investigated for different *N* values. Figure 5 shows maximum gain variation versus injection current values for different numbers of QD groups. That the horizontal axis indicates Δ*I* (and not *I*) is the delicate point here. It is presumed that sufficient time has been spent which is vital for occupation probabilities to reach their steady-state condition before applying the input optical power pulse. Through this, the structure has required some *I*_0_ crucial for reaching this condition; thus, the initial optical gain value is not zero, and the horizontal axis is described in terms of Δ*I* = *I* − *I*_0_. Evidently, as the injection current reaches higher points, the energy states will be filled with higher carrier densities, which in turn results in higher optical gain values. Furthermore, as *N* increases, the superimposition effect will be more intense, and the maximum gain value will increase as well. This could be a useful tool for engineering the optical gain peak in high-power applications.

### Customized Gain

**Figure 6:**
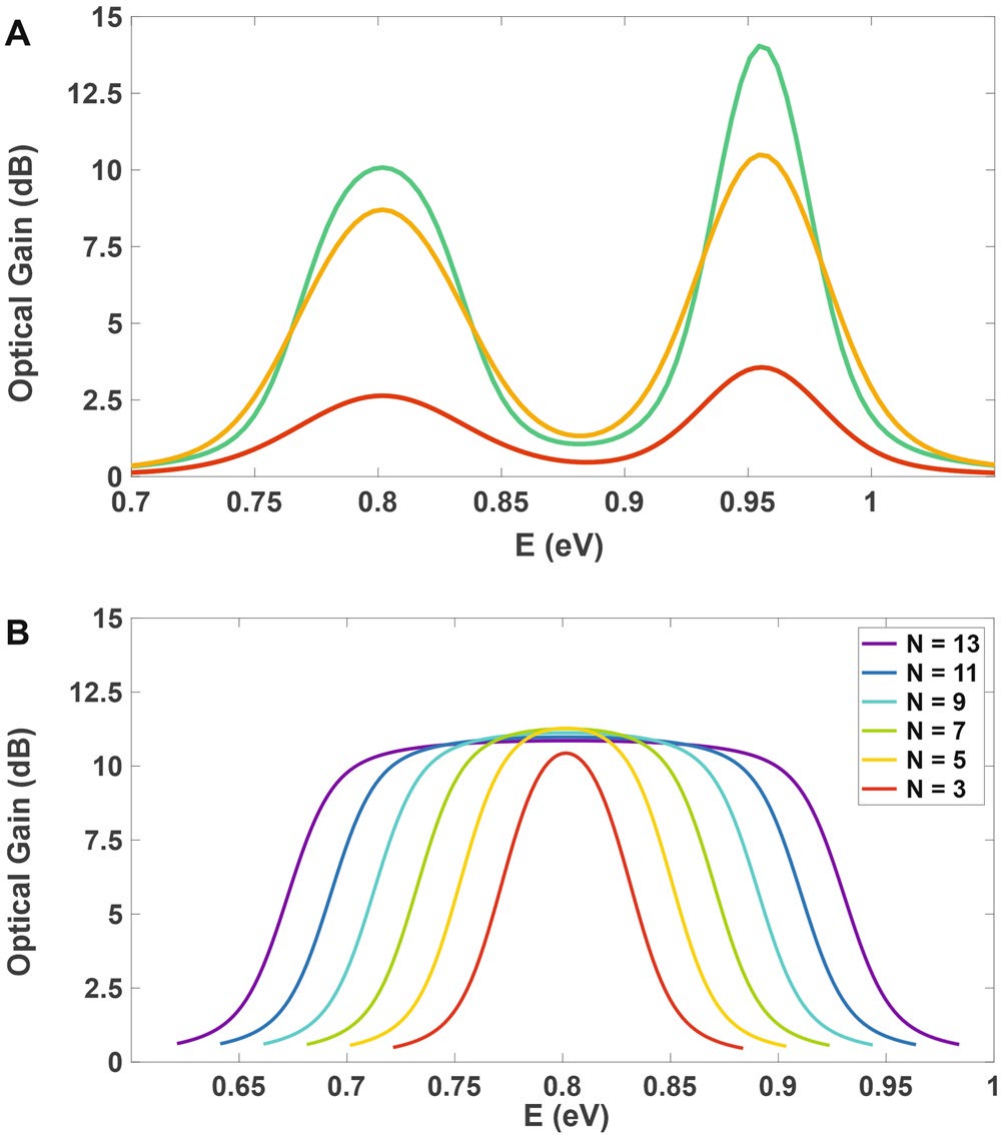
Customized optical gain spectrum and optical gain broadening. (**A**) Customized gain spectrum for two optical windows over 1.55 μm and 1.31 μm with respectively ${\varGamma }_{{G}_{1}}$ = 74 meV and ${\varGamma }_{{G}_{2}}$ = 51 meV for *N* = 1 (red); the optical gain peak values for this setting is 3 dB and 4 dB. In two other cases for which ${\varGamma }_{{G}_{1}}$ = ${\varGamma }_{{G}_{2}}$ = 30 meV (green), ${\varGamma }_{{G}_{1}}$ = ${\varGamma }_{{G}_{2}}$ = 50 meV (orange), and *N* = 3, the optical gain peak values are 10 dB and 14 dB for *Γ*_*G*_ = 30 meV and 9 dB and 11 dB for *Γ*_*G*_ = 50 meV, (**B**) broadened gain profiles over 1.55 µm for 3, 5, 7, 9, 11, and 13 groups of QDs.

Stated above, our model has the flexibility to produce optical gain output for customized features as in Fig. 3(B). Considering the special case consisting two optical gain windows, 1.5 μm–1.6 μm and 1.25 μm–1.35 μm, our model has been able to result in almost 3 dB and 4 dB optical gain peak values with *N* = 1 for respectively *Γ*_*G*_ = 74 meV and 51 meV. The resultant optical gain spectrum is shown in Fig. 6(A) for which the gain peak values can be tuned to special applications using different *N* and QD volume densities in the structure. The optimum case in which *N* = 3 and *Γ*_*G*_ = 30 meV and another one with *N* = 3 and *Γ*_*G*_ = 50 meV are also drawn in Fig. 6(A) for better comparisons with the first one; optical gain peak values are 10 dB and 14 dB for *Γ*_*G*_ = 30 meV and 9 dB and 11 dB for *Γ*_*G*_ = 50 meV. Clearly, one can obtain other desired optical gain spectra by tuning *N* and *Γ*_*G*_. Practically, the customized gain output for *N* = 1 is obtained, but in order to achieve higher gain peak values, higher *N* values can be possibly used.

### Optical Gain broadening investigation

As the main objective of this letter, optical gain broadening over various *N* values has been investigated in Fig. 6(B). The figure demonstrates that one can have broader optical gain spectra increasing *N* and considering more intense superimposition effect caused by more QD groups. Obviously from the figure, the optical gain spectrum is illustrated for *N* = 3, 5, 7, 9, 11, and 13; increasing *N* has led the optical gain bandwidth to broaden almost from 0.12 μm to 0.52 μm. This bandwidth can be broadened more incorporating higher *N* values for wider applications, among which one was drawn in Fig. 4(A) using *N* = 5 and *Γ*_*G*_ = 300 meV covering more than 1.02 μm optical bandwidth. As mentioned earlier, the bandwidth is not restricted to this limit; it can be manipulated by setting appropriate values of required parameters for the desired cases. Also, our concentration in this paper was on only one type of material; it is well obvious that using multiple types of materials, the desired broadband spectrum can be obtained without limitation.

## Conclusion

In this letter, an ultra-broadband quantum dot semiconductor optical amplifier (QD-SOA) considering the solution-processed technology was proposed with more than 1.02 μm optical gain bandwidth covering *O*, *C*, *S*, and *L* bands, just using a single type of material. The bandwidth, which can be broadened more to span wider spectral ranges for specific applications, was obtained using the proposed superimposition model, which controls some features of QDs to reach the desired optical gain spectrum. Also, considering the solution-processed technology and choosing suitable synthesis methods, nanoparticles with a given precision and resolution can be fabricated, and the proposed method for gain engineering is possible to realize. Managing the number of QD groups, the structure has the capability to produce ultra-broadband optical gain output as a result of the superimposition effect. Furthermore, by controlling their size distribution over a specific QD radius and FWHM of inhomogeneous broadening, it could produce customized optical gain profiles over desired optical windows, among which a widely-applicable case was demonstrated here for 1.31 μm and 1.55 μm. More on the gain characteristics, the structure was analyzed for some factors affecting the optical gain profile. The injection current directly impacted the optical gain peak value. The effect of changing FWHM for homogeneous and inhomogeneous broadenings on the optical gain output was also discussed in the letter. Finally in this work, a design procedure for optical broadband gain spectrum was proposed, and a realization method based on solution-processed technology was presented.
